# Incorporation of Glutamic Acid or Amino-Protected Glutamic Acid into Poly(Glycerol Sebacate): Synthesis and Characterization

**DOI:** 10.3390/polym14112206

**Published:** 2022-05-29

**Authors:** Yi-Sheng Jiang, Ming-Hsien Hu, Jeng-Shiung Jan, Jin-Jia Hu

**Affiliations:** 1 Department of Chemical Engineering, National Cheng Kung University, Tainan 70101, Taiwan; js3794490@gmail.com; 2 Bachelor Program for Design and Materials for Medical Equipment and Devices, Da-Yeh University, Changhua 515, Taiwan; minghsienhu@gmail.com; 3 Orthopedic Department, Showchwan Memorial Hospital, Changhua 500, Taiwan; 4 Hierarchical Green-Energy Materials (Hi-GEM) Research Center, National Cheng Kung University, Tainan 70101, Taiwan; 5 Department of Mechanical Engineering, National Yang Ming Chiao Tung University, Hsinchu 300, Taiwan

**Keywords:** poly(glycerol sebacate), poly(ester amide), elastomer, lipase, π-π stacking interactions, pi stacking, soft tissue engineering

## Abstract

Poly(glycerol sebacate) (PGS), a soft, tough elastomer with excellent biocompatibility, has been exploited successfully in many tissue engineering applications. Although tunable to some extent, the rapid in vivo degradation kinetics of PGS is not compatible with the healing rate of some tissues. The incorporation of L-glutamic acid into a PGS network with an aim to retard the degradation rate of PGS through the formation of peptide bonds was conducted in this study. A series of poly(glycerol sebacate glutamate) (PGSE) containing various molar ratios of sebacic acid/L-glutamic acid were synthesized. Two kinds of amino-protected glutamic acids, Boc-L-glutamic acid and Z-L-glutamic acid were used to prepare controls that consist of no peptide bonds, denoted as PGSE-B and PGSE-Z, respectively. The prepolymers were characterized using ^1^H-NMR spectroscopy. Cured elastomers were characterized using FT-IR, DSC, TGA, mechanical testing, and contact angle measurement. *In vitro* enzymatic degradation of PGSE over a period of 28 days was investigated. FT-IR spectroscopy confirmed the formation of peptide bonds. The glass transition temperature for the elastomer was found to increase as the ratio of sebacic acid/glutamic acid was increased to four. The decomposition temperature of the elastomer decreased as the amount of glutamic acid was increased. PGSE exhibited less stiffness and larger elongation at break as the ratio of sebacic acid/glutamic acid was decreased. Notably, PGSE-Z was stiffer and had smaller elongation at break than PGSE and PGSE-B at the same molar ratio of monomers. The results of *in vitro* enzymatic degradation demonstrated that PGSE has a lower degradation rate than does PGS, whereas PGSE-B and PGSE-Z degrade at a greater rate than does PGS. SEM images suggest that the degradation of these crosslinked elastomers is due to surface erosion. The cytocompatibility of PGSE was considered acceptable although slightly lower than that of PGS. The altered mechanical properties and retarded degradation kinetics for PGSE reflect the influence of peptide bonds formed by the introduction of L-glutamic acid. PGSE displaying a lower degradation rate compared to that for PGS can be used as a scaffold material for the repair or regeneration of tissues that are featured by a low healing rate.

## 1. Introduction

Tissue engineering integrates cells, biodegradable scaffolds, growth/stimulating factors or their combinations to restore, maintain, or improve damaged tissues or whole organs [1]. Specifically, the scaffold, before being replaced by the cell-produced extracellular matrix, serves as a substrate for cell adhesion and subsequently a microstructural guideline for tissue development. Tremendous efforts have been made to synthesize biodegradable polymers that can be used to fabricate scaffolds for tissue engineering applications.

Polyester is one of most frequently used biodegradable polymers in tissue engineering. Thermoplastic polyesters such as poly(lactic acid) (PLA), poly(glycolic acid) (PGA), polycaprolactone (PCL) and their copolymers have been widely used in biomedical applications due to their biocompatibility, biodegradability and ease of processing [2]. The mechanical properties of PGA, PLA, PCL and their copolymers are not always appropriate for use in the repair or regeneration of some tissues, however [3,4]. Crosslinked polyesters such as poly(glycerol sebacate) (PGS) [5], poly(1,8-octanediol citrate) [6], on the other hand, have several superior features for use as scaffold materials including, particularly, a tunable degradation rate and mechanical properties.

PGS is a biodegradable elastomer synthesized by condensation of glycerol and sebacic acid [5], both of which are bio-based materials and approved by US FDA for medical applications. As glycerol has three hydroxyl groups and sebacic acid has two carboxyl groups, their reaction results in a three-dimensional crosslinked polymeric network, which explains the toughness and elasticity of PGS. PGS has been used successfully in a variety of tissue engineering applications due to its excellent biocompatibility, ease of synthesis, surface-erosion biodegradability, rubberlike elasticity, and tunable mechanical properties [7,8,9,10,11].

PGS scaffolds are conventionally fabricated by solvent casting followed by curing and salt leaching. Mechanically anisotropic PGS scaffolds have been prepared using microfabrication techniques [12] or aligned sacrificial fibers [13,14]. Fibrous PGS scaffolds that mimic the extracellular matrix of tissues can be prepared by blend or coaxial electrospinning [15,16,17,18]. Recently, high-molecular-weight PGS prepolymers are synthesized by lipase-catalyzed esterification and used to prepare electrospun nanofibers [19,20]. More information about PGS can be found in several reviews [21,22,23,24].

PGS is resorbed completely within 60 days after subcutaneous implantation in rats [25]. The fast in vivo degradation kinetics is inconsistent with *in vitro* degradation studies involving no enzymes [5,26]. The rapid degradation of PGS, which is independent of crosslink density [27], could represent a limitation, however, and could limit its use as a scaffold material for the repair or regeneration of tissues that require a long healing time such as cardiac muscle.

The functional groups of glycerol and sebacic acid can react with those of other monomers and thus offer unlimited possibilities for the generation of new crosslinked polymers for specific purposes. For example, the introduction of lactic acid into a PGS network modulates the microstructure of PGS analogues, and hence the degradation rate and mechanical properties [28], whereas the introduction of glycolic acid increases the degradation rate [29]. With the introduction of citric acid, PGS analogues can be crosslinked under relatively mild conditions [30,31]. Polyethylene glycol may be integrated similarly into a PGS network for improved hydrophilicity and hence better cytocompatibility [32,33]. The introduction of Boc-L-glutamic acid increases the hydrophilicity and degradation rate for poly(xylitol glutamate sebacate) [34]. PGS has been endowed with antimicrobial capacity by grafting L-arginine [35]. Photocurable PGS prepolymer has been prepared using methacrylic anhydride to be used in additive manufacturing [36]. Besides reactions involving functional groups, PGS composites can be formed by crosslinking blends of PGS prepolymer and other functional molecules. For example, PGS prepolymer and polyaniline have been blended and crosslinked to form electrically conductive composites for cardiac tissue engineering [37]. 

Poly(ester amide)s that integrate the excellent thermal and mechanical properties of polyamides with the biocompatibility and biodegradability of polyesters have gained increasing attention for medical applications, including use as scaffolds for tissue engineering [38,39,40]. In this study, L-glutamic acid was incorporated into a PGS network with an aim to retard the degradation rate of PGS through the formation of peptide bonds. L-Glutamic acid has two carboxyl groups and one amino group; the former can react with hydroxyl groups in PGS prepolymer to form ester bonds and the latter with carboxyl groups to form peptide bonds. Two kinds of amino-protected glutamic acid, Boc-L-glutamic acid and Z-L-glutamic acid, were also used to prepare control materials that consist of no peptide bonds for comparison.

## 2. Materials and Methods

### 2.1. Materials

Glycerol, dimethyl sulfoxide-d_6_ (DMSO-d_6_) and tetramethylsilane were purchased from Sigma-Aldrich (St. Louis, MO, USA). Sebacic acid was purchased from ACROS (St. Louis, MO, USA). L-glutamic acid was purchased from Alfa Aesar (Ward Hill, MA, USA). Boc-L-glutamic acid and Z-L-glutamic acid were purchased from Bachem (Bubendorf, Switzerland). Acetone andtetrahydrofuran (THF) were purchased from Macron (Center Valley, PA, USA). For cell culture, Dulbecco’s modified Eagle’s medium (DMEM), penicillin/streptomycin, and trypsin-EDTA were obtained from Thermo Fisher Scientific (Waltham, MA, USA) and calf serum was purchased from Hyclone (Logan, UT, USA). WST-8 assay was purchased from Abbkine (Wuhan, China). All chemicals were used as received without further purification.

### 2.2. Synthesis and Characterization of Prepolymers

**Scheme 1:**
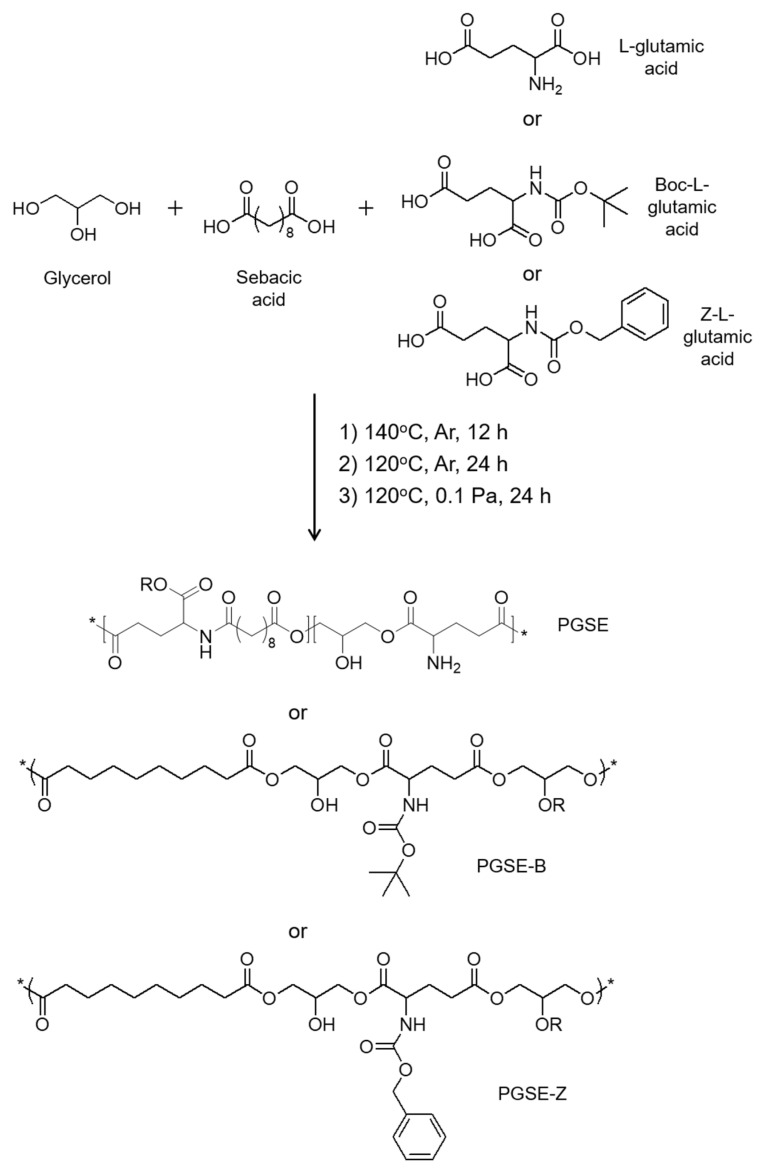
Synthesis of the prepolymers.

Prepolymers for poly(glycerol-sebacate-glutamate) (PGSE), poly(glycerol-sebacate-Boc-glutamate) (PGSE-B), poly(glycerol-sebacate-Z-glutamate) (PGSE-Z) were prepared based on the method developed by Wang et al. [5] with modifications (Scheme 1). Prescribed amounts of glycerol, sebacic acid and L-glutamic acid (or Boc-L-glutamic acid or Z-L-glutamic acid) (Table 1) were reacted with agitation under nitrogen at 140°C in a 250 mL three-neck flask connected to a Dean–Stark trap for 12 h. The temperature was reduced to 120°C and continued for another 24 h. The pressure of the flask was reduced to 0.1 Pa and continued at 0.1 Pa and 120°C for another 24 h to obtain the prepolymer. PGS prepolymer was prepared similarly as a control. ^1^H NMR was used to characterize the chemical structure of the prepolymers. The prepolymers were dissolved in DMSO-d_6_ to prepare 20 mg/mL solutions. ^1^H NMR spectra were recorded on an Avance 600 MHz NMR spectrometer (Bruker; Billerica, MA, USA). Chemical shifts were given in ppm with tetramethylsilane as a standard. Gel permeation chromatography was performed to estimate the molecular weight of the prepolymers. A Shimadzu modular system with an autosampler and a differential refractive index detector was used. Three Shodex LF-604 columns (6.0 mm × 150 mm; Showa Denko, Tokyo, Japan) were employed in series. The prepolymers were dissolved in THF to prepare 3 mg/mL solutions, which were filtered before injection. The mobile phase was THF at 40°C with a flow rate of 0.3 mL/min. Calibration was achieved by monodisperse polystyrene standards ranging from 1.31 to 71.8 kg/mol.

### 2.3. Preparation of Elastomers

To prepare elastomer films from prepolymers, the prepolymer was first dissolved in acetone to make a 30% (*w*/*v*) solution. The solution was poured into a PTFE mold. Acetone was then removed by placing the mold on a 60°C heater. Upon the removal of acetone, the prepolymer in the mold was cured in a vacuum oven at 150°C under 0.1 Pa for 48 h.

### 2.4. Fourier-Transform Infrared Spectroscopy

The chemical structure of the cured PGS, PGSE, PGSE-B, and PGSE-Z was examined by infrared spectroscopy. Fourier transform infrared (FT-IR) spectra of the elastomers were obtained by scanning the specimens for 64 times in the wave number range from 600–4000 cm^−1^ at a resolution of 1 cm^−1^ using a Nicolet Nexus 670 FT-IR spectrometer (Thermo Fisher Scientific, Waltham, MA, USA) with an ATR module.

### 2.5. Thermal Properties

Differential scanning calorimetry (DSC) was performed with a Q20 differential scanning calorimeter (TA Instruments, New Castle, DE, USA). Samples of PGS, PGSE, PGSE-B, and PGSE-Z weighing ~3 mg were used. The experiments were conducted at a heating rate of 10 °C/min from −60 to 150°C. The sample chamber was purged with nitrogen gas at a rate of 15 mL/min. The thermal stability of the elastomers was examined by a TGA 4000 Thermogravimetric Analyzer (Perkin Elmer, Waltham, MA, USA). Samples weighing ~12 mg were subjected to a ramp rate of 10 °C/min from 25 to 600°C. The decomposition temperature is defined as the temperature at which the remaining weight reaches 95% of the initial dry weight.

### 2.6. Mechanical Testing

Uniaxial tensile testing was performed using a custom-made mechanical tester [41] equipped with a 1000-g load cell (WMCP-1000G, Interface, Scottsdale, AZ, USA). Dog-bone shaped specimens were punched from the elastomeric film using a miniature ASTM D412-C die (gauge length: 16.5 mm; width: 3 mm). The specimen was stretched to failure at a strain rate of 0.03 min^−1^. The deformation of the specimen was assessed by tracking the positions of twelve markers attached on the central region of the specimen with respect to their positions in the unloaded configuration and presented in terms of stretch ratio in the stretching direction. The thickness of the specimen was measured by the high-frequency ultrasound system. Given the deformation and the thickness of the specimen, Cauchy stress can be calculated based on tensile force recorded by the load cell. From the stress–stretch curve of the specimen, the Young’s modulus, the ultimate strength, and the elongation at break were determined (*n* = 5–6).

### 2.7. Water Contact Angle Measurements

The wettability of PGS, PGSE, PGSE-B and PGSE-Z films was assessed by water contact angle measurements. Water drops of 5 µL were carefully deposited on the surface of the specimens by a micro-pipette. Images of the droplets on the surface were acquired by a digital camera (Canon EOS 850D, Tokyo, Japan) and the contact angles were measured by ImageJ (NIH, USA) with an average of 6 measurements for each group.

### 2.8. In Vitro Enzymatic Degradation

Disk samples of diameter of 6 mm were punched out of ~500-µm thick elastomeric films. The disk was weighed (giving a weight W_day 0_) and sterilized with 70% ethanol followed by drying under UV irradiation in a laminar flow hood overnight. The disk was immersed in a 1.5-mL lipase solution (600 U/mL) and incubated at 37°C in a CO_2_ incubator (NuAire, Plymouth, MN, USA). After incubation for 2, 7, 14, 21, and 28 days, the disk was removed, washed with deionized water, and dried under vacuum. After weighing (giving a weight W_day i_), the disk was placed in a new lipase solution. The remaining weight percentage was calculated using the following formula:

(1) Wremaining(%)=Wday iWday 0×100%

where *W_day i_* represents the dry weight on day *i*; *i*= 2, 7, 14, 21, or 28, *W_day 0_* is the initial dry weight. The surface morphology of the sample was examined on day 0, 7, and 24 by scanning electron microscopy. Pieces of dry samples were mounted onto stubs, sputter-coated with platinum (Sputter E-1045, Hitachi, Japan), and viewed in a scanning electron microscope (S-4100, Hitachi, Japan).

### 2.9. Cytocompatibility

NIH/3T3 cells were cultured in direct contact with the elastomer specimens to assess the *in vitro* cytocompatibility of the specimens. Briefly, the cells were seeded in a 96-well plate at a density of 2500/well. Upon cell adhesion, 20-mg specimens were added into the cell-seeded wells. The cells were co-cultured with the specimen for two or four days prior to the WST-8 assay, which was performed following a standard protocol; living cells reduce WST-8 in the serum-free medium, resulting in color change of the medium. The absorbance of the medium containing reduced or unreacted WST-8 was evaluated at 450 nm using a FlexStation 3 microplate reader (Molecular Devices, Sunnyvale, CA, USA). The viability of the cells was presented as OD_sample—_OD_blank_ where OD_sample_ and OD_blank_ are the absorbance of the test wells containing WST-8 reduced by the cells that were cultured with the specimen and that containing unreacted WST-8, respectively. At least 4 specimens for each group were analyzed. The cells after the WST-8 assay were fixed by 4% formaldehyde and immune-stained for GAPDH (primary antibody: rabbit anti-mouse GAPDH; secondary antibody: FITC conjugated goat anti-rabbit IgG). Cell images were acquired using a fluorescence microscope (CKX53, Olympus, Japan) equipped with a CMOS camera (DP28, Olympus, Japan).

### 2.10. Statistical Analysis

Data are presented as mean ± standard deviation. Differences in mechanical properties and cytocompatibility were analyzed by one-way ANOVA in conjunction with Tukey post hoc procedure. The level of significance was set at 0.05.

## 3. Results and Discussion

**Figure 1:**
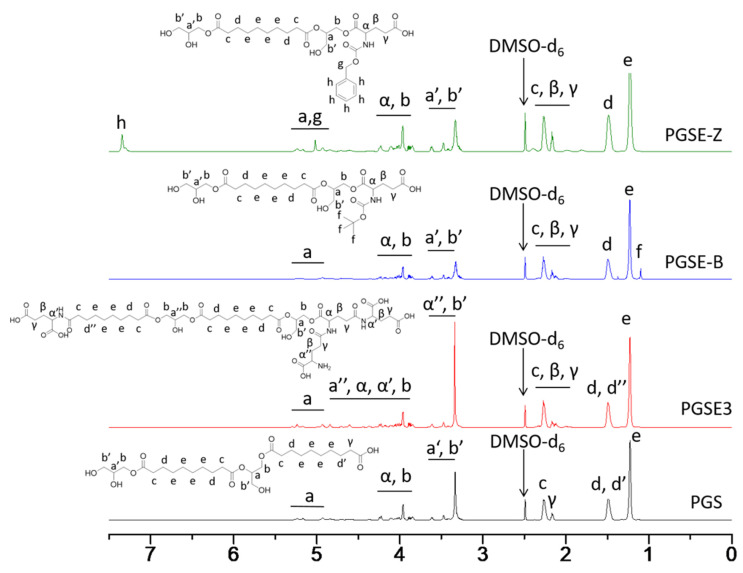
^1^H-NMR of the prepolymers for PGS, PGSE3, PGSE-B, and PGSE-Z.

The structure of the prepolymers of PGS, PGSE3, PGSE-B, and PGSE-Z was analyzed by ^1^H-NMR spectroscopy. Figure 1 shows the ^1^H-NMR spectra of the prepolymers with each peak assigned. The peaks at δ 1.23, 1.48, and 2.26 ppm suggested that sebacic acid was included in all of the prepolymers. The peaks at δ 3.3–4.4 ppm indicated protons on unesterified glycerol, whereas the peaks at δ 4.6-5.3 ppm indicated protons on esterified glycerol. PGSE-B had a unique peak at δ 1.38 ppm and PGSE-Z had another unique peak at δ 7.34 ppm, which indicated the proton in the *tert*-butyl group and benzene, respectively. Note that the crosslinking reactions occurred randomly and it is not realistic to predict the exact structure of the prepolymers.

The molecular weight of the prepolymers are listed in Table 2. The molecular weight of PGSE2 prepolymer was slightly higher than that of others, probably because of more reactants were involved. On the other hand, the lowest molecular weight of PGSE-B prepolymer may be attributed to the presence of the steric bulk in Boc-L-glutamic acid. Note that PGSE-Z prepolymer had an unusually high PDI (i.e., broad molecular weight distribution) compared with other prepolymers. This may be due to π-π stacking interactions between the benzene rings associated with Z-glutamic acid.

**Figure 2:**
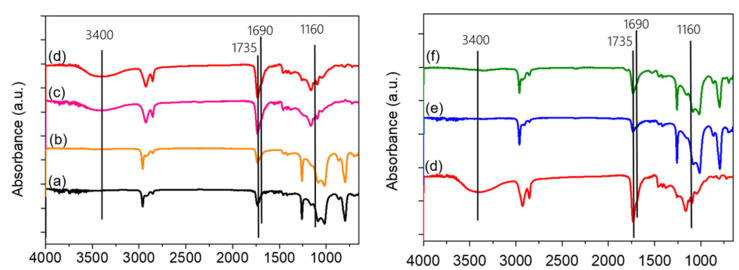
ATR-FTIR spectra of PGS (**a**), PGSE1 (**b**), PGSE2 (**c**), PGSE3 (**d**), PGSE-B (**e**) and PGSE-Z (**f**).

The structure of the cured PGS, PGSE3, PGSE-B, and PGSE-Z was analyzed by FT-IR spectroscopy and the results are shown in Figure 2. The absence of hydroxyl group in PGS implied that all the three hydroxyl groups in glycerol were reacted. The broad band at 3400 cm^−1^ in the spectra of PGSE2 and PGSE3 indicated secondary amine stretching; notably, PGSE1 did not display the peak and neither did PGSE-B and PGSE-Z. The peak at 1160 cm^−1^ attributed to the C-N stretching of amide was obvious in PGSE2 and PGSE3. The peak at 1735 cm^−1^ associated with the C=O stretching of ester was observed in all the elastomers. PGSE2 and PGSE3 exhibited a peak at 1690 cm^−1^, which corresponded to the C=O stretching of amide. The results suggested the formation of peptide bonds between L-glutamic acid and sebacic acid.

**Figure 3:**
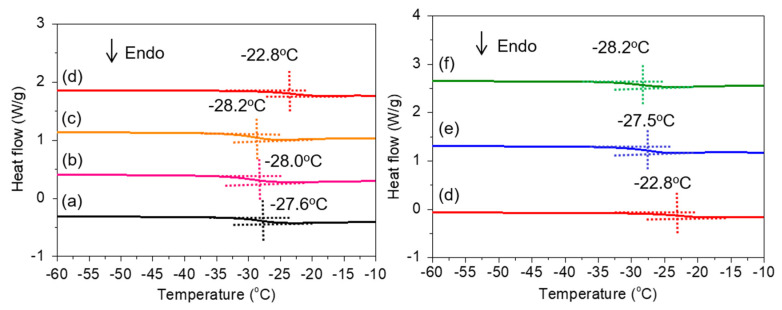
DSC heating curves of PGS (**a**), PGSE1 (**b**), PGSE2 (**c**), PGSE3 (**d**), PGSE-B (**e**), and PGSE-Z (**f**).

DSC was used to examine the glass transition temperature of PGS, PGSE1, PGSE2, PGSE3, PGSE-B, and PGSE-Z. Figure 3 shows DSC heating curves of the elastomers. The glass transition temperature (*T*_g_) of all the elastomers was below room temperature, which indicates that all the elastomers are in a rubbery state at room temperature or under physiological conditions. PGSE1 and PGSE2 had a slightly lower *T*_g_ compared to PGS. The reduction in *T*_g_ might be attributed to the compromised crystallinity of the prepolymer caused by the addition of a small amount of L-glutamic acid. Notably, further reducing the molar ratio of sebacic acid/glutamic acid significantly increased the *T*_g_; PGSE3 had a significantly higher *T*_g_ compared to PGS. Furthermore, it should be noted that PGSE-B and PGSE-Z had a *T*_g_ comparable to PGS.

**Figure 4:**
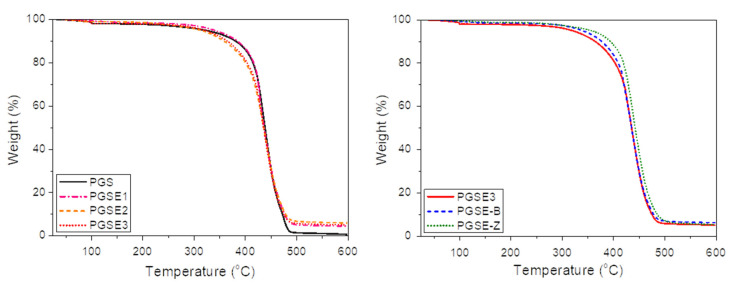
TGA curves of PGS, PGSE1, PGSE2, PGSE3, PGSE-B, and PGSE-Z.

Figure 4 shows the results of thermogravimetric analysis of the elastomers. The partial substitution of sebacic acid with glutamic acid appeared to compromise the thermal stability of the elastomer as PGSE had a lower decomposition temperature compared to PGS. The decomposition temperature decreased with decreasing ratio of sebacic acid/glutamic acid given the same amount of carboxyl groups; that is, PGSE3 < PGSE1. On the other hand, the decomposition temperature, given the same molar ratio of monomers (1: 0.8: 0.2), was in the order: PGSE3 ≅ PGSE-B < PGSE-Z.

**Figure 5:**
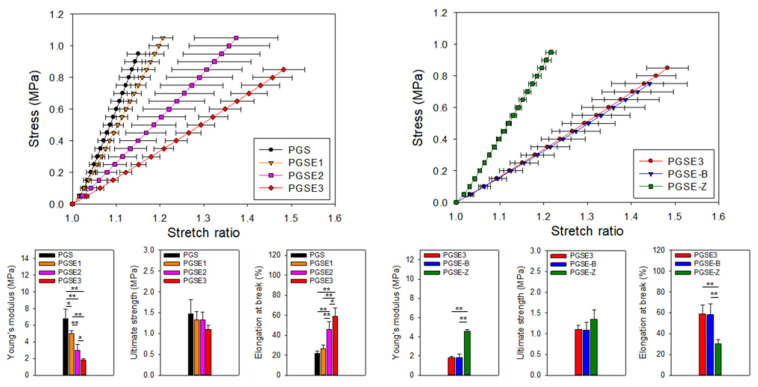
Average stress–stretch curves and mechanical properties of PGS, PGSE1, PGSE2, PGSE3, PGSE-B, and PGSE-Z (* means p<0.05 and ** means p<0.001).

The mechanical behaviors of the elastomers are shown in Figure 5. For PGSE, the Young’s modulus decreased and the elongation at break increased with the decreasing ratio of sebacic acid/glutamic acid, whereas the ultimate strength did not change significantly. Notably, given the same molar ratio of monomers, PGSE-Z was stiffer and had a smaller elongation at break than PGSE and PGSE-B, presumably due to the π-π stacking interactions between the benzene rings associated with Z-glutamic acid [42,43].

**Figure 6:**
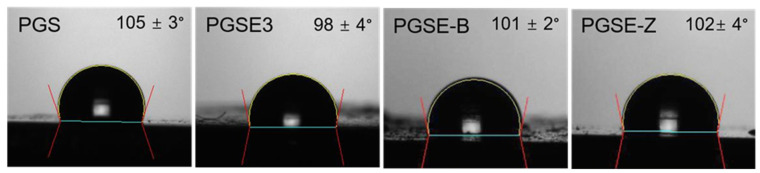
Representative photographs of water droplets on the surface of PGS, PGSE3, PGSE-B, and PGSE-Z and their associated contact angles (*n* = 6).

Scaffold materials for tissue engineering should have proper surface hydrophilicity for cells to adhere. Figure 6 shows the water contact angle of the elastomers. The incorporation of glutamic acid slightly increased the hydrophilicity of PGSE; PGSE3 had the smaller contact angle than PGS. Both PGSE-B and PGSE-Z had a contact angle comparable to PGS.

**Figure 7:**
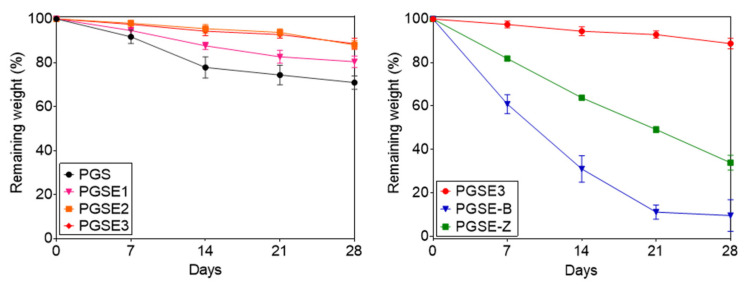
In vitro enzymatic degradation of PGS, PGSE1, PGSE2, PGSE3, PGSE-B, and PGSE-Z.

**Figure 8:**
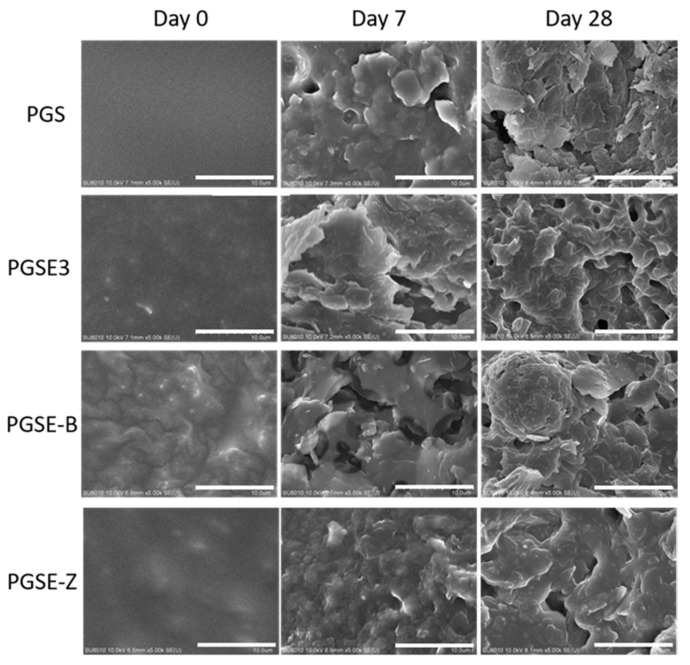
SEM images of surface morphology of PGS, PGSE3, PGSE-B, and PGSE-Z on days zero, seven, and twenty-eight.

Past studies showed that PGS was completely degraded 42 to 60 days after implantation in rats [44,45]. To better mimic the physiological conditions in which PGSE is degraded, the degradation of PGSE in a lipase solution was investigated [27]. Lipase is one of the important enzymes for fat metabolism and capable of hydrolyzing ester bonds. The results of *in vitro* enzymatic degradation are shown in Figure 7. PGSE had a lower degradation rate than PGS; increasing the amount of glutamic acid reduced the degradation rate of PGSE. Note that the degradation rate of PGSE2 and PGSE3 was comparable probably due to the same amount of glutamic acid involved in the synthesis and hence similar density of peptide bonds. PGSE-B and PGSE-Z that consisted of no peptide bonds degraded faster than PGSE3 given the same molar ratio of monomers and also faster than PGS, which suggests the positive effects of peptide bonds on retarding the degradation of PGSE. The SEM images shown in Figure 8 illustrated that the degradation of these crosslinked elastomers was due to surface erosion. In this study, the *in vitro* degradation was performed with the elastomers in the form of solid films. The degradation of porous scaffolds may be different due to the presence of porosity. Recently, the porosity and curing temperature were found to affect the PGS degradation kinetics [46].

**Figure 9:**
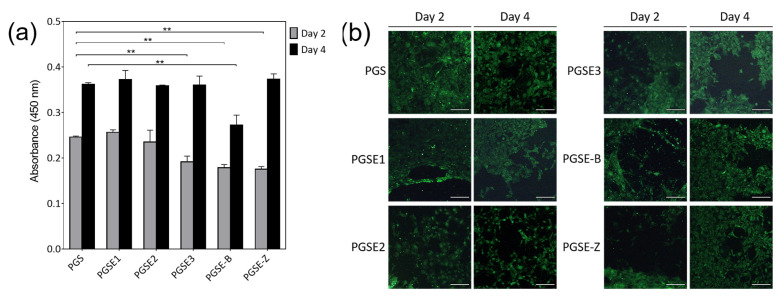
(**a**) Growth of NIH/3T3 co-cultured with PGS, PGSE1, PGSE2, PGSE3, PGSE-B, or PGSE-Z (** means *p* < 0.001). (**b**) Representative immunofluorescence images of the corresponding cells stained for GAPDH (scale bar = 100 μm).

The cytocompatibility of PGS, PGSE1, PGSE2, PGSE3, PGSE-B, and PGSE-Z is shown in Figure 9. On day two, the viability of the cells cultured with PGSE3, PGES-B, and PGSE-Z was less than that of PGS. The reduced cytocompatibility may be attributed to unreacted L-glutamic acid, Boc-L-glutamic acid, or Z-L-glutamic acid that could be leached into the medium during the test. The molar ratio of the reactants tested in this study might not be ideal, which could lead to soluble portion of the elastomers. Nevertheless, unreacted L-glutamic acid could be leached from PGSE before its use. Interestingly, the viability of the cells cultured with PGSE3 and PGSE-Z returned to the level comparable to that of PGS on day four. The immunofluorescence images stained for GAPDH suggest that culturing with the materials did not affect cell morphology.

PGSE developed in this study can be used to repair or regenerate soft tissues that are subjected to a mechanically active environment and have a low healing rate such as cardiac muscles. It can also be exploited in the development of a drug delivery platform for the cases in which a longer release profile is desired.

## 4. Conclusions

PGSE that contains peptide bonds in a crosslinked network was successfully synthesized by melt polycondensation of glycerol, sebacic acid, and L-glutamic acid. The incorporation of L-glutamic acid resulted in a network that was less stiff and had larger elongation at break. Notably, PGSE-Z was stiffer and had a smaller elongation at break than PGSE and PGSE-B, presumably due to π-π stacking interactions. The results of *in vitro* enzymatic degradation demonstrated that PGSE has a lower degradation rate than does PGS whereas PGSE-B and PGSE-Z degrade at a greater rate than does PGS. The cytocompatibility of PGSE was considered acceptable although slightly lower than that of PGS. The altered mechanical properties and retarded degradation kinetics of PGSE reflect the influence of peptide bonds formed by the introduction of L-glutamic acid. We conclude that PGSE can be used as a scaffold material for the repair or regeneration of tissues that are featured by a low healing rate.

## Figures and Tables

**Table 1:** Feed ratio of the prepolymers.

Sample Code	Composition	Molar Ratio
PGS	glycerol: sebacic acid	1: 1
PGSE1	glycerol: sebacic acid: L-glutamic acid	1: 0.9: 0.1
PGSE2	glycerol: sebacic acid: L-glutamic acid	1: 0.9: 0.2
PGSE3	glycerol: sebacic acid: L-glutamic acid	1: 0.8: 0.2
PGSE-B	glycerol: sebacic acid: Boc-L-glutamic acid	1: 0.8: 0.2
PGSE-Z	glycerol: sebacic acid: Z-L-glutamic acid	1: 0.8: 0.2

**Table 2:** Molecular weight of the prepolymers.

Sample Code	Molecular Weight and Its Distrubution (Mw/Mn/PDI)
PGS	5261 / 2192 / 2.4
PGSE1	5468 / 2497 / 2.2
PGSE2	10177 / 3002 / 3.4
PGSE3	5574 / 2362 / 2.4
PGSE-B	2243 / 1089 / 2.1
PGSE-Z	15604 / 2123 / 7.4

## Data Availability

The data presented in this study are available on request from the corresponding author.
